# Airborne transmission of common swine viruses

**DOI:** 10.1186/s40813-023-00346-6

**Published:** 2023-10-31

**Authors:** Zhiqiang Hu, Xiaogang Tian, Ranran Lai, Chongxing Ji, Xiaowen Li

**Affiliations:** 1 Shandong Engineering Laboratory of Pig and Poultry Healthy Breeding and Disease Diagnosis Technology, Xiajin New Hope Liuhe Agriculture and Animal Husbandry Co., Ltd, Xiajin Economic Development Zone, Qingwo Venture Park, Dezhou, 253200 Shandong Province People’s Republic of China; 2 Key Laboratory of Feed and Livestock and Poultry Products Quality and Safety Control, Ministry of Agriculture and Rural Affairs, New Hope Liuhe Co., Ltd, 316 Jinshi Road, Chengdu, 610100 Sichuan People’s Republic of China; 3 Shandong New Hope Liuhe Co., Ltd, No. 592-26 Jiushui East Road Laoshan District, Qingdao, 266100 Shandong People’s Republic of China; 4 Shandong New Hope Liuhe Agriculture and Animal Husbandry Technology Co., Ltd (NHLH Academy of Swine Research), 6596 Dongfanghong East Road, Yuanqiao Town, Dezhou, 253000 Shandong People’s Republic of China; 5 China Agriculture Research System-Yangling Comprehensive Test Station, Intersection of Changqing Road and Park Road 1, Yangling District, Xianyang, People’s Republic of China

**Keywords:** Aerosol, Swine virus, Transmission distance, Experimental condition, Field condition, Influential factors, Preventive strategy

## Abstract

The transmission of viral aerosols poses a vulnerable aspect in the biosecurity measures aimed at preventing and controlling swine virus in pig production. Consequently, comprehending and mitigating the spread of aerosols holds paramount significance for the overall well-being of pig populations. This paper offers a comprehensive review of transmission characteristics, influential factors and preventive strategies of common swine viral aerosols. Firstly, certain viruses such as foot-and-mouth disease virus (FMDV), porcine reproductive and respiratory syndrome virus (PRRSV), influenza A viruses (IAV), porcine epidemic diarrhea virus (PEDV) and pseudorabies virus (PRV) have the potential to be transmitted over long distances (exceeding 150 m) through aerosols, thereby posing a substantial risk primarily to inter-farm transmission. Additionally, other viruses like classical swine fever virus (CSFV) and African swine fever virus (ASFV) can be transmitted over short distances (ranging from 0 to 150 m) through aerosols, posing a threat primarily to intra-farm transmission. Secondly, various significant factors, including aerosol particle sizes, viral strains, the host sensitivity to viruses, weather conditions, geographical conditions, as well as environmental conditions, exert a considerable influence on the transmission of viral aerosols. Researches on these factors serve as a foundation for the development of strategies to combat viral aerosol transmission in pig farms. Finally, we propose several preventive and control strategies that can be implemented in pig farms, primarily encompassing the implementation of early warning models, viral aerosol detection, and air pretreatment. This comprehensive review aims to provide a valuable reference for the formulation of efficient measures targeted at mitigating the transmission of viral aerosols among swine populations.

## Background

The significance of biosecurity in farms is increasingly acknowledged due to the significant challenges posed by the ASFV to the global pig industry, particularly in Asia, where conventional interventions such as vaccines or drugs have proven ineffective in addressing these issues [1, 2]. In the biosecurity system, aerosol transmission has always been a weak link in biosecurity prevention and control, thus understanding and preventing aerosols is extremely important for the health of pig populations. The investigation of aerosol transmission is a specialized and noteworthy area of study, particularly in relation to the examination of swine viral aerosols. In a previous review, STARK [3] presented a comprehensive overview of pathogen aerosols in pig farms, offering valuable and enduring perspectives that continue to be cited and employed by farmers, despite the passage of time. However, recent advancements in research techniques and the growing needs of farmers have led to the identification of additional aerosol-borne viruses and the revelation of more intricate transmission patterns.

Bioaerosols, characterized by the presence of small droplets or particles measuring less than 5 μm suspended in a gaseous medium, possess the capacity to transport pathogenic microorganisms and facilitate the transmission of diseases [4]. The present review is specifically dedicated to viral aerosols laden with swine viruses. Respiratory activity in swine populations serves as a notable origin of aerosols [5]. Pathogenic viruses can be actively or passively released into the air via aerosols, resulting in extensive dissemination of the virus [6–8]. The potential of viral aerosols to induce diseases is primarily contingent upon the infectivity of the pathogen and the requisite dosage for a susceptible host [9]. The dynamics and dispersion of viral aerosols are influenced by a multitude of factors, including aerosol diameter, initial velocity, temperature, relative humidity, ultraviolet radiation, airflow, ventilation, and filtration [10–12]. The viability of viruses within aerosols is impacted by the initial metabolic state of the virus, genetic characteristics, and the surrounding environment.

This review comprehensively summarizes the latest characteristics of aerosol transmission for various common swine viruses (Table 1), including FMDV, PRRSV, IAV, PEDV, PRV, CSFV, ASFV, porcine circovirus (PCV), swine vesicular disease virus (SVDV), Japanese encephalitis virus (JEV), and porcine respiratory coronavirus (PRCV). Notably, it encompasses a summary of several recently discovered viruses that exhibit the ability to be transmitted via aerosols, including SIV, JEV, PCV, and PEDV. Additionally, by drawing upon practical experiences and strategies employed in the prevention and control of human aerosol-transmitted viruses, we propose innovative approaches that hold potential for application in the prevention and control of aerosol transmission within pig production. This review is expected to serve as a valuable reference for the formulation of effective strategies to prevent the transmission of viral aerosols among pig populations.

**Table 1:** Characteristics and influential factors of aerosol transmission for different swine viruses

Category	Virus	Diameter (nm)	Distance of transmission	Viral loads in aerosols	Experimental evidence	Field evidence	Influential factors
Long-distance	FMDV	26	300 km [18, 19]	10^5.8^–10^6.4^TCID_50_ [24]	Yes [24]	Yes [17, 19]	Viral strain, host species, weather condition, geographical condition, environmental condition, contract structure
	PRRSV	60	9.2 km [30]	6 × 10^2^–5.1 × 10^4^copies /m^3^ [35]	Yes [28, 29]	Yes [30]	Viral strain, mixed pathogen, aerosol particle size, environmental condition
	IAV	80–120	2.1 km [40]	10^4^–10^7^ copies/m^3^ [39]	Yes [41]	Yes [40]	Viral strain, aerosol particle size, environmental condition,
	PEDV	95–190	16.1 km [51]	1.3 × 10^6^–3.5 × 10^8^copies/m^3^ [35]	Yes [54]	Yes [51]	Viral strain, aerosol particle size, wind direction, age of susceptible animals
	PRV	225	13.8 km [60]	10^5.3^ TCID_50_ [59]	Yes [59]	Yes [60]	Unknown
Short-distance	CSFV	40–50	1 m [65]	10^1.2^–10^3.8^TCID_50_/m^3^ [70]	Yes [65]	Yes [64]	Viral strain, viral dose, RH, wind speed
	ASFV	200	10 m [78]	10^3.2^ TCID_50_/m^3^ [81]	Yes [76, 77]	Yes [78]	Unknown
Others	PCV	17	Unknown	10^7^ copies/m^3^ [84]	Unknown	Yes [84]	Unknown
	SVDV	24–26	Unknown	10^1.4^–10^2.6^ TCID_50_ [89]	Unknown	Yes [89]	Unknown
	JEV	50	Unknown	Unknown	Yes [97]	Unknown	Unknown
	PRCV	100–160	Unknown	10^1.87^PFU/m^3^ [104]	Yes [105]	Unknown	Unknown

### Airborne swine viruses in pigs

In this section, we summarize the characteristics and influential factors of aerosol transmission of various prevalent swine viruses. The transmission distance of viral aerosols holds significant importance, as evidenced by a scholarly report suggesting that a spatial separation exceeding 150 m between structures can effectively mitigate the risks associated with airborne transmission [3]. Consequently, we classify these viruses into three distinct categories based on their respective aerosol transmission distances (Table 1): long-distance transmission viruses (exceeding 150 m), short-distance transmission viruses (ranging from 0 to 150 m), and other viruses with unknown transmission distances. The long-distance transmission viruses primarily occur between farms, while the short-distance transmission viruses mainly occur within a farm.

### Long-distance airborne viruses

#### FMDV

FMDV, belonging to the *Aphthovirus* genus within the *Picornaviridae* family, is an enveloped, single-stranded positive-sense RNA virus with a diameter of approximately 26 nm. It primarily causes vesicular diseases affecting the oral mucosa, hoof parts, and udder skin in pigs, cattle, and sheep, which is also a zoonotic disease [13]. The airborne transmission of FMDV under filed conditions has been extensively investigated, with a particular focus on the creation of mathematical models that incorporate variables such as meteorological conditions and wind patterns [14–16]. These models have greatly expanded our understanding of the characteristics of FMDV airborne transmission. Hugh-Jones’ report indicates that aerosol transmission of FMDV on land can reach distances of 60–150 km with the assistance of favorable weather conditions, including wind and rain [17]. Moreover, the transportation of viruses over long distances through plumes is particularly probable across seaways due to the minimal surface turbulence and the ability to sustain airborne particle concentrations for greater distances compared to land areas [18]. It is believed that the farthest distance of airborne dissemination over the sea is approximately 300 km, from pig farms experiencing FMDV outbreaks in Brittany (northern France) to cattle farms located on the Isle of Wight [14, 17, 19]. Consequently, the capacity for FMDV to transmit through the air is influenced by both weather conditions and geographical factors. Another important factor is that susceptibility to the virus varies among different species, with ruminants becoming infected with as little as 10 TCID50 dose, whereas pigs require a dose of 6 × 10^3^ TCID50 [20, 21]. And the shed virus in saliva and nasal swabs from FMDV-infected pigs is 100–1000 times more infectious for sheep and cattle than the minimum infectious dose, indicating the potential role of pigs as an important source for airborne transmission [22, 23]. An integrated model developed by SéRENSEN et al. showed that when 1,000 pigs were infected and released the virus, the longest airborne distances for downwind cattle, sheep and pigs were 300 km, 90 km and 20 km, respectively, which also supports the above opinion [15]. Under experimental conditions, more details about aerosol propagation signatures of FMDV were discovered. In a study by Alexandersen et al., three strains of FMDV in aerosols showed that each pig could release 10^5.8^–10^6.4^ TCID50 of the virus into the air within a 24-h period [24]. Eble et al. conducted a study and found that the estimated between-pen transmission rate of FMDV was 0.59 per day, while the within-pen transmission rate was 6.14. This suggests that the contact structure between pigs significantly influences the transmission rate of FMDV [25]. Furthermore, it has been observed that FMDV has a greater likelihood of survival in environments with temperatures below 60 °C, relative humidity (RH) above 55%, and a neutral pH. This indicates that environmental factors may also have a significant impact on the airborne transmission of FMDV [26].

#### PRRSV

PRRSV, belonging to the *Arterivirus* genus within the *Arteriviridae* family, is an enveloped, single-stranded positive-sense RNA virus with a diameter of approximately 60 nm. It primarily causes reproductive disorders in sows and respiratory difficulties in piglets [27]. Research has demonstrated that PRRSV can be transmitted through aerosols with a range of 0.5–150 m under experimental conditions [28, 29], whereas the distance can extend up to 9.2 km or even further under field conditions [30]. The primary factors effecting the airborne transmission capacity of PRRSV are viral strains and mixed pathogens. On the one hand, Torremorell et al. found that the PRRSV strain VR-2332 could be transmitted through the air, while highly pathogenic field strains (MN-1b) could not [31]. Research conducted by Cho et al. also demonstrated that the pathogenicity of PRRSV isolates significantly affects the frequency of aerosol shedding but does not significantly influence the concentration of the virus in aerosols [32, 33]. On the other hand, Otake et al. demonstrated that airborne transmission only occurred under mixed conditions with PRRSV variants 1-8-4 and porcine pneumonia-associated *Mycoplasma hyopneumoniae* (MHYO) 232, and the air samples collected at a distance of 9.2 km from the pig population remained infectious [30]. Additionally, Brockmeier et al. reported that pigs co-infected with PRRSV and *Actinobacillus pleuropneumoniae* exhibited an increased frequency and duration of sneezing and coughing, indicating a higher potential for aerosol transmission of pathogens [34]. Other important factors are aerosol particle sizes and environmental conditions. Alonso et al. demonstrated that the concentration of PRRSV viral particles in aerosols with different diameters range from 6 × 10^2^ (0.4–0.7 μm) to 5.1 × 10^4^ (9.0–10.0 μm) copies/m^3^ [35]. The half-life of PRRSV in aerosols is less than 30 min at a temperature of 30 °C, and low RH conditions favor the survival of the virus in aerosols [2, 36], which is the basis for developing measures for virus aerosol disinfection in farms.

#### IAV

IAV, belonging to the *Influenza A* genus within the *Orthomyxoviridae* family, is an enveloped single-stranded negative-sense RNA virus with a diameter of 80–120 nm. It primarily causes a zoonotic respiratory disease characterized by coughing, respiratory distress, and fever [37]. The airborne transmission has been detected under both experimental conditions and field conditions. Under field conditions, a study revealed that 71% of the 122 bioaerosol samples collected from large-scale farms tested positive for IAV [38]. And, IAV was isolated from indoor aerosol samples of a commercial pig farm, with the highest viral loads observed 7–11 days after an outbreak (10^4^–10^7^ copies/m^3^), persisting for 20 days [39]. Furthermore, IAV has been isolated from air samples collected both inside and outside pig farms [40], indicating the risk of airborne transmission between farms. Further studies showed that the transmission distance has been observed to be up to 2.1 km downwind [40]. Under experimental conditions, Zhang et al. demonstrated IAV of S-O 2009 IV strain was able to be aerosolized by infected animals and to be transmitted to susceptible animals by airborne routes in pig and guinea infection models, and the airborne distance was a range of 2.0–4.2 m [41].

There is a limited amount of literature available regarding the aerosol transmission factors impacting swine influenza virus. Nevertheless, there exists a substantial body of research on other species that can be utilized as a point of reference. The primary factors influencing the capacity of IAV airborne transmission are viral strains, environmental conditions, and sizes of aerosol particles. Mubareka et al. [42] discovered notable variations in the aerosol transmission capacity among different strains of IAV, and subsequent investigations have confirmed that these differences can be attributed to amino acid mutations in the neuraminidase and hemagglutinin gene of different IAV strains, which can affect the affinities between virus and receptors of different hosts and viral replication ability in the respiratory tract [43–45]. Environmental conditions influence airborne transmission mainly by RH and ambient air temperature [46]. Under field conditions, research has also found that environmental conditions with a RH of 20–35% and a temperature of 5 °C are most favorable for IAV transmission [47]. Furthermore, Lindsley et al. [48] conducted a study employing a human cough model, which revealed the presence of IAV RNA in aerosol particles of various sizes. The findings indicated that 35% of the RNA was detected in particles larger than 4 µm, 23% in particles ranging from 1 to 4 µm, and 42% in particles smaller than 1 µm. These particles fell within the respirable size range.

#### PEDV

PEDV, belonging to the *Coronavirus* genus within the *Coronaviridae* family, is an enveloped, single-stranded positive-sense RNA virus with a diameter of approximately 95-190 nm. It primarily causes severe enteric diseases with high mortality and watery diarrhea in 7-day-old piglets [49, 50]. Aerosol transmission of PEDV has been observed up to a distance of 16.1 km under field conditions, as first detected by Alonso et al. in infectious viral particles in aerosol samples from PEDV-positive pig herds [51]. The aerosol transmission of PEDV is primarily related to aerosol particle size, viral strains, wind direction, and the age of susceptible animals [52]. PEDV can be detected in aerosol particles across all diameter ranges, with the contents from 1.3 × 10^6^ (0.4–0.7 μm) to 3.5 × 10^8^ (9.0–10.0 μm) copies/m^3^, and the aerosols with viral particles can survive in the environment for up to 9 months [35, 53]. Research by Gallien et al. showed that under experimental conditions, non-InDel PEDV strains exhibited earlier detection and higher viral loads in the air under experimental conditions, indicating a higher efficiency of aerosol transmission [54]. Furthermore, Beam et al. utilized geospatial methods and meteorological data to establish a correlation between wind direction and the aerosol transmission of PEDV [55]. Li's research provided evidence of pre-weaning piglets being infected with PEDV through viral aerosols, as intranasal administration of 1 mL of wild-type PEDV (10^7^ PFU/mL) resulted in typical PED symptoms in 5-day-old piglets [56]. However, Niederwerder et al. found that aerosols generated from PEDV-inoculated animals did not cause disease in 4-week-old piglets, suggesting that pigs of different ages may have varying tolerance to viral aerosols [57].

#### PRV

PRV, belonging to the *Varicellovirus* genus within the *Herpesviridae* family, is an enveloped double-stranded DNA virus with a diameter of approximately 225 nm. It primarily causes an acute infectious disease characterized by high fever, abortion, and neurological symptoms not only in pigs, domestic livestock, and various wild animals [58]. Donaldson et al. conducted an experiment under experimental conditions wherein healthy pigs in one pen were effectively infected through exposure to aerosols emitted by PRV-positive pigs housed in another pen, connected by a pipe [59]. Each PRV-positive pig released virus particles into the air, with peak titers reaching a maximum of 10^5.3^ TCID50 within 24 h [59]. Grant et al. further substantiated the aerosol transmission of PRV through the utilization of a Gaussian diffusion model [60]. Under field conditions, aerosols containing PRV can be dispersed by the wind, reaching distances ranging from 1.3 to 13.8 km or even more [60–62]. However, there is currently a lack of in-depth study on the airborne transmission of PRV.

### Short-distance airborne viruses

#### CSFV

CSFV, belonging to the genus *Pestivirus* within the *Flaviviridae* family, is an enveloped, single-stranded positive-sense RNA virus with a diameter of 24–26 nm. It mainly causes acute fever and bleeding in pigs [63]. A study by Elbers et al. analyzed factors related to the field transmission of CSFV and suggested airborne transmission as one of the most important routes of infection [64]. Gonzalez et al. demonstrated that CSFV can be transmitted through aerosols in non-contact situations under an experimental condition, with a transmission distance of up to 1 m. The main factors affecting airborne transmission of CSFV include RH, wind speed, viral dose, and viral strains [65]. Lu et al. confirmed that low relative humidity and high wind speed are important factors contributing to CSFV outbreaks, as they promote the widespread dissemination of aerosols [66]. Two studies by Laevens et al. showed that CSFV was only transmitted through aerosols to other pig pens after all the pigs in the infected pen have become ill, suggesting that a certain level of viral loads is required for aerosol transmission [67, 68]. Weesendorp et al. found that airborne transmission played an important role in the later stage of CSF outbreaks, which aligns with Laevens’ conclusions [69]. Additionally, Weesendorp et al. further studied the airborne transmission of different CSFV strains with varying virulence and found that strains with higher viral concentrations or higher virulence were associated with higher viral loads in aerosol samples, ranging from 10^1.2^ to 10^3.0^ TCID50/m^3^ (moderate virulence) to 10^1.6^–10^3.8^ TCID50/m^3^ (high virulence) [70]. Moreover, research has shown that CSFV remains infectious in an aerosolized state for at least 30 min, with a half-life ranging from 4.5 to 15 min [71].

#### ASFV

ASFV, belonging to the *Asfivirus* genus within the *Asfarviridae* family, is an enveloped double-stranded DNA virus with a diameter of approximately 200 nm. It is also the only known DNA arthropod-borne virus [72, 73]. ASFV primarily causes an acute, hemorrhagic, highly contagious disease in domestic and wild pigs, with a mortality rate of up to 100% [74, 75]. Wilkinson et al. demonstrated that ASFV can be transmitted by aerosols from ASFV African strains-positive pigs, with a potential distance infecting up to 2.3 m, providing initial evidence of airborne transmission of ASFV under experiment conditions [76]. European ASFV strains were also shown to spread through aerosols among pigs through aerosols, infecting healthy pigs in separate pens [77]. According to the latest research by Li et al., the transmission distance of ASFV Asian strains under field conditions can reach up to 10 m [78]. And secretions carrying high viral titers from ASFV-positive pigs during sneezing and coughing can be aerosolized and emitted into the environment [79, 80]. Under experimental conditions, the half-life of ASFV in aerosols was approximately 14 min, with viral titers up to 10^3.2^ TCID50/m^3^ found in aerosol samples collected from rooms with ASFV-positive pigs [81]. The prevention of aerosol transmission of ASFV constitutes a crucial component of prevailing biosecurity systems implemented in numerous pig farming operations. Regrettably, the present body of research on the mechanism, determinants, and preventive strategies pertaining to aerosol transmission remains insufficient. Given the persistent threat posed by ASFV to the worldwide swine industry, it is imperative that this area be urgently explored through future investigations.

### Other viruses with unknown transmission distances

#### PCV

PCV, belonging to the *Circovirus* genus within the *Circoviridae* family, is a non-enveloped single-stranded DNA virus with a diameter of approximately 17 nm. It primarily targets and impairs the immune system of susceptible animals [82, 83]. Verreault et al. discovered the presence of PCV2 in the air of pig barns, with a concentration of 10^7^ copies/m^3^ [84]. Another study also detected PCV2-positive aerosol samples in pig farms and slaughterhouses [85]. Additionally, PCV2 was found in nasal lavage samples from farmers (4/78) working in a pig farm, indicating a high risk of PCV2 transmission through aerosols [86]. However, the transmission distance and the precise mechanisms of aerosol transmission of PCV2 are not yet clear.

#### SVDV

SVDV, belonging to the *Enteroviru*s genus within the *Picornaviridae* family, is a non-enveloped, single-stranded positive-sense RNA virus with a diameter of approximately 24–26 nm. It primarily causes vesicular diseases affecting the hooves, oral cavity, nostrils, and mammary glands in pigs [87, 88]. Under field conditions, Sellers et al. collected aerosol samples from SVDV-positive pig herds and detected SVDV viral particles in aerosols of different particle sizes, ranging from 10^1.4^ TCID50 (< 3 μm) to 10^2.6^ TCID50 (> 6 μm) [89]. SVDV viral particles were also found in the noses of farmers who had been in contact with pigs for more than 5 min, with viral titers approximately 10^2.4^ TCID50 [89]. It is suspected that the main source of aerosols containing SVDV is the shedding of virus particles from ruptured lesions which subsequently form aerosols, leading to rapid transmission [90]. And SVDV can be detected in aerosols within 2–3 days after infection, with viral loads being 160 times lower than those of FMDV [23, 91]. However, there is currently a lack of in-depth study on the airborne transmission of PRV.

#### JEV

JEV, belonging to the *Flavivirus* genus within the *Flaviviridae* family, is an enveloped, single-stranded positive-sense RNA virus with a diameter of approximately 45–50 nm. It primarily causes a zoonotic disease characterized by high fever and neurological symptoms [92]. JEV transmission has been exclusively described as being mosquito-mediated. However, studies have shown that the nasal mucosa serves as an entry and exit route for the virus [93, 94]. In an animal experiment, the average virus titer in the nasal secretions of infected pigs was 2.25 × 10^2^ TCID50/mL [95], and even a low virus titer of 10 TCID50/mL successfully infected pigs through the intranasal route [96]. The aforementioned findings suggest that pigs are susceptible to infection by aerosols carrying JEV. Thus far, only one study has provided evidence of aerosol transmission of JEV between mice under experimental conditions [97].

#### PRCV

PRCV, belonging to the *Coronavirus* genus within the *Coronaviridae* family, is an enveloped, single-stranded positive-sense RNA virus with a diameter of approximately 100–160 nm. It is a natural deletion mutant of the enteropathogenic TGEV [98–100]. PRCV primarily causes subclinical respiratory symptoms, such as mild bronchio-interstitial pneumonia and neutrophil infiltration [101, 102]. Costantini et al. have demonstrated that PRCV can replicate in the respiratory tracts of pigs and be transmitted through aerosols, infecting pigs of any age through contact or airborne transmission [103]. Research by Bourgueil et al. in experimental conditions also demonstrated that air samples from pig pens with PRCV-positive pigs remained positive for 6 days, with the highest level reaching 10^1.87^ PFU/m^3^ [104]. Cox et al. aerosolized solutions containing the PRCV-TLM83 strain at a concentration of 10^7^ TCID50, and successfully infected 1-week-old piglets by inoculating them with the collected aerosols [105]. Another research by Keep et al. indicated that there was no difference of viral load detection and pathology in respiratory tissues of PRCV-infected pigs with two different infection routes, aerosol and intranasal/tracheal, which indicating a high sensitivity of PRCV-positive aerosols by pigs [106]. It is worth noting that PRCV is highly sensitive to environmental factors and can only be sustained under specific conditions, namely 47% relative humidity and a temperature of 20 °C [107]. Currently, there is a lack of research on the transmission distance and other comprehensive investigations of PRCV aerosols.

Above all, the primary evidence concerning the transmission of viral aerosols is derived from field and laboratory studies. Detecting aerosol transmission under field conditions, especially in the early stages, poses significant challenges. Mathematical models based on epidemiological surveys and case reports play a crucial role in understanding long-distance virus transmission and the emergence of new viruses. Extensive research has been conducted on the utilization of mathematical models in the context of FMDV [14–16], and this approach can be further applied to the investigation of other virus aerosols. Moreover, recent comprehensive case reports and source tracing analyses pertaining to the aerosol transmission of ASFV [78] and COVID-19 [108] have yielded exemplary demonstrations. Consequently, more case reports on the aerosol transmission of viruses should be encouraged in the future. Furthermore, the transmission of viral aerosols in pig farms is significantly influenced by several key factors, including aerosol particle sizes, viral strains, the host sensitivity to viruses, weather conditions, geographical conditions and environmental conditions, which supply basis for the development against viral aerosols in pig farms.

### Prevention of airborne viruses in pig farms

Airborne transmission plays a pivotal role in the containment of animal diseases and is equally significant in the dissemination of zoonotic diseases. Consequently, it is imperative to prioritize endeavors aimed at preventing airborne transmission. However, given the scarcity of research on the prevention of aerosol transmission in pig farms and the current reliance on relatively simplistic preventive measures, here, we propose integrating insights from studies on viral aerosol transmission diseases in humans to propose potential preventive and control strategies that could be implemented in pig farms.

### Establishment of early warning models

The implementation of a real-time early warning monitoring system or regional risk maps for viral aerosol transmission is crucial for disease prevention, control and the improvement of biosecurity measures for pig farms. The establishment of an early warning model for West Nile virus provides a valuable reference [109]. Firstly, it is important to note that the long-distance transmission of most aerosol-borne diseases, such as FMDV [17, 19] and PEDV [55], is strongly influenced by climate and geographical factors, as previously mentioned. Hence, climate factors including temperature, humidity, precipitation, wind speed, and wind direction, as well as geographical factors such as altitude and terrain, should be included as parameters within this model. Secondly, the inclusion of epidemiological investigation data pertaining to previous viral diseases within a particular region is crucial. Lastly, it is imperative to incorporate routine pathogen monitoring data pertaining to personnel, drinking water, wastewater, feed, and manure in pig farms as variables in this model, as they can serve as indicators for evaluating the extent of environmental contamination in a specific locality. For example, Silva's report highlights the significance of wastewater monitoring in enhancing the early warning surveillance of SARS-CoV-2 transmission [110]. By collecting the aforementioned parameters, an early warning model can be established, which represents interdisciplinary research that holds significant implications for the pig farming industry.

### Detection of viral aerosols

Monitoring viral aerosols in pig farms can function as a proactive measure to mitigate airborne transmission [111]. However, the efficacy of air sampling and pathogen detection is significantly diminished under field conditions due to high gas exchange rates. Consequently, enhancing the sensitivity of detection methods and the collection efficiency of air samplers can enhance the likelihood of identifying viral aerosols during the early stages of transmission. Given the escalating threat of ASFV to pig production, numerous detection methods for swine viruses with high sensitivity have been devised, such as digital polymerase chain reaction (dPCR) [112], insulated Isothermal PCR (iiPCR) [113], offering promising prospects for detection of viral aerosols. As for air sampling devices, Li et al. used two different air samplers with different flow rates to collect aerosols within and between barns respectively, demonstrating the aerosol transmission of ASFV under field conditions [78]. In a separate study, Lee et al. developed a portable integrated bioaerosol sampling/ monitoring platform that can detect viral aerosol particles within 20 min using the signal of near-infrared (NIR)-to-NIR nanoprobes [114]. Moreover, Yao et al. have successfully developed an ultrasensitive and rapid “sample-to-answer” microsystem for on-site monitoring of SARS-CoV-2 in aerosols, which has been effectively implemented in various public settings including airports and hospitals [115]. The mobility, high sensitivity, and capability of these aforementioned techniques to capture aerosols in open spaces align precisely with the needs of contemporary pig farms. The potential integration of such devices within pig farms would undoubtedly enhance the efficacy of viral aerosol detection.

### Air pretreatment

The process of air pretreatment involves the elimination of pathogens from the air prior to its entry into the barn, with the aim of ensuring its safety. A viable approach entails the implementation of air filtration systems to purify the air entering pig barns, a strategy that has been extensively employed in global large-scale pig farms, demonstrating its efficacy in disease control, particularly in the case of PRRS [116, 117]. However, the cost and maintenance requirements of air filtration systems make them affordable only for large-scale farming enterprises. An alternative, more cost-effective method is air disinfection. Previous studies have demonstrated the efficacy of chlorine dioxide aerosol as a disinfectant in fitness centers [118], and it has also shown promising results in the disinfection of ASFV, IAV, and Mycobacterium [119, 120]. Hence, the utilization of chlorine dioxide as a means of air pre-treatment in pig barns exhibits considerable promise. Furthermore, the exploration of chlorine dioxide's potential as a sustained-release formulation for ongoing disinfection within the barn presents a viable option. Additionally, studies have indicated that ions produced by electrostatic disinfectors and ozone gas demonstrate efficacy in disinfecting and managing airborne aerosols containing SARS-CoV-2 [121, 122], thereby suggesting their potential applicability in pig farming settings.

### Others

Other methods, such as the reduction of dust levels in pig barns and the establishment of disease-free zones, hold potential for the prevention and control of viral aerosols [2, 8]. Additionally, nasal vaccination, which can elicit both humoral and cellular immune responses, offers effective protection against respiratory pathogens, as the nasal cavity serves as the primary entry route for airborne pathogens [123]. Consequently, nasal vaccination presents a promising approach for attaining optimal protection against respiratory pathogens.

## Conclusion

**Fig. 1:**
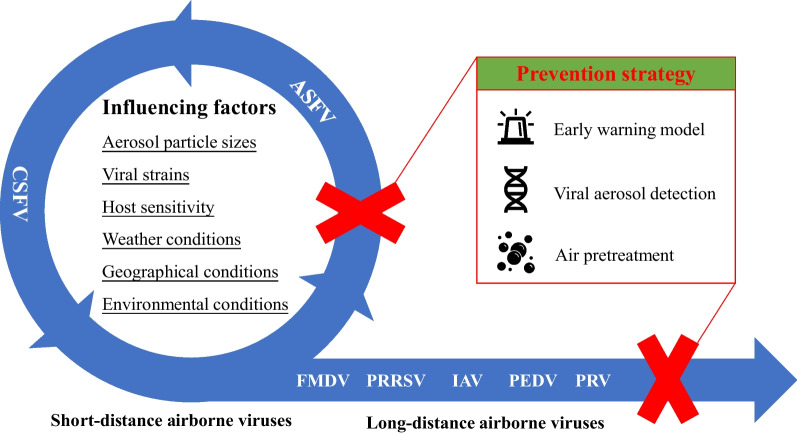
Schematic diagram of aerosol transmission and prevention for swine viruses

In conclusion, this review provides a systematic classification and summary of prevalent airborne-transmitted viruses in pig production. As shown in Fig. 1, it encompasses the transmission characteristics, influential factors, and preventive strategies, with the objective of offering valuable references and innovative perspectives for mitigating and managing aerosol transmission. The significance of early-warning systems for viral aerosols, the enhancement of detection sensitivity and air sampler collection efficiency, as well as the development of air pretreatment strategies, are emphasized as crucial measures to establish a low-risk environment with fresh air for pig herds in the future.

## Data Availability

Not applicable.

## Abbreviations

FMDV: Foot-and-mouth disease virus
PRRSV: Porcine reproductive and respiratory syndrome virus
IAV: Influenza A viruses
PEDV: Porcine epidemic diarrhea virus
PRV: Pseudorabies virus
CSFV: Classical swine fever virus
ASFV: African swine fever virus
PCV: Porcine circovirus
SVDV: Swine vesicular disease virus
JEV: Japanese encephalitis virus
PRCV: Porcine respiratory coronavirus
MHYO: *Mycoplasma hyopneumoniae*
RH: Relative humidity
dPCR: Digital polymerase chain reaction
iiPCR: Insulated isothermal PCR
NIR: Near-infrared
